# Early Warning Value of ASL-MRI to Estimate Premorbid Variations in Patients With Early Postoperative Cognitive Dysfunctions

**DOI:** 10.3389/fnagi.2021.670332

**Published:** 2021-08-17

**Authors:** Xue Du, Yan Gao, Su Liu, Jingya Zhang, Diksha Basnet, Junjun Yang, Jiehui Liu, Yijie Deng, Jiayong Hu, Peijun Wang, Jianhui Liu

**Affiliations:** ^1^Department of Anesthesiology, Tongji Hospital, School of Medicine, Tongji University, Shanghai, China; ^2^Department of Radiology, Tongji Hospital, School of Medicine, Tongji University, Shanghai, China

**Keywords:** arterial spin labeling, magnetic resonance imaging, postoperative cognitive dysfunction, cerebral blood flow, pre-operative changes in brain

## Abstract

**Background**: Postoperative cognitive dysfunction (POCD) is a general complication following cardiac and major non-cardiac surgery amongst the elderly, yet its causes and mechanisms are still unknown. The present study aimed to detect whether regional cerebral blood flow (CBF) is altered in the brain before surgery in POCD patients compared with non-POCD (NPOCD) patients, thus, CBF variation may potentially predict the occurrence of early POCD.

**Methods**: Fifty patients scheduled for spinal stenosis surgery were enrolled in the study. All study participants completed a battery of neuropsychological tests (NPTs) by a well-trained neuropsychologist before the surgery and 1 week after the surgery. POCD was defined when the preoperative to postoperative difference of at least two of the NPTs’ |Z|-scores with reference to a control group exceeded 1.96. Pulsed arterial spin-labeling (ASL) MRI was scanned at least 1 day before surgery. The ASLtbx toolkit and SPM12 were applied to preprocess and correct the images, which were then normalized to the MNI brain template space to obtain standardized cerebral perfusion images.

**Results**: POCD was identified in 11 out of 50 patients (22%). The CBF of the right superior temporal lobe, right and left middle cingulate gyrus, and the right hippocampus, and parahippocampal gyrus in POCD group was lower than that in NPOCD group (*P* < 0.001). The CBF of the pars triangularis of inferior frontal gyrus in POCD group was higher than that in NPOCD group (*P* < 0.001).

**Conclusions**: These preliminary findings suggest that CBF premorbid alterations may happen in cognitively intact elderly patients that develop early POCD. Alterations of preoperative CBF might be a bio-marker for early POCD that can be detected by noninvasive MRI scans.

## Introduction

Surgical procedures and general anesthesia are correlated to a large number of complications, including postoperative cognitive dysfunction (POCD) or delayed neurocognitive recovery. Particularly in elderly patients, the POCD incidence after surgery is high, ranging from 10% to 62% (Chi et al., 2017; Glumac et al., 2019), and POCD seriously affects the mental health, social aspects, and quality of life of the aged patients (Spalletta et al., 2012; Needham et al., 2017). The main POCD susceptibility factors include aging combined with diabetes mellitus, hypertension, coronary heart disease, and other comorbidities, type and time of operation, anesthesia type, and anesthetic methods (Czyz-Szypenbejl et al., 2019). It can last for several days to several months (Rundshagen, 2014). POCD has a considerable impact on the healthcare system, which can lead to prolonged hospital admission, reduced quality of life, and increased dependency (Williams-Russo et al., 1995; Moller et al., 1998; Newman et al., 2001; Monk et al., 2008). To apply preventative measures and better balance the surgery risks and benefits, it is of great importance to identify more accurately preoperative patients at increased POCD risk. Neuropsychological tests (NPTs) are regarded to be salient among objective methods of cognition assessment. A complete cognitive function assessment is fairly difficult, and conducting a full neuropsychological examination is tedious and straining for patients. Therefore, it is urgent to search for an objective and effective tool to evaluate the POCD.

Advancements in neuro-imaging, especially in magnetic resonance imaging (MRI) have provided helpful tools to noninvasively measure neuronal activity as well as capture detailed human brain images (Alexopoulos et al., 2012). The present study tested the hypothesis that arterial spin-labeling (ASL) neuro-imaging might depict early and subtle changes in brain perfusion preoperatively in cognitively intact elderly patients and this information could be utilized to predict early phases of subsequent cognitive decline (Xekardaki et al., 2015). The major advantages of ASL MRI are its non-invasiveness without contrast agent injection and the lack of exposure to ionizing radiation (Riederer et al., 2018). MRI could be employed to establish neuropathological POCD aspects. MRI results relevant to POCD could help clinicians diagnose patients at high risk and reduce postoperative mortality (Kant et al., 2017). To date, there is still a lack of evidence of ASL-associated POCD diagnosis. The present study aimed to search and identify ASL findings associated with those cognitive deficits to help prediction and prevention of POCD patients at high risk.

## Materials and Methods

### Participants

A total of 103 patients scheduled for spinal stenosis surgery were admitted to Tongji Hospital from January 2017 to December 2019 and were screened for the study. Written informed consent was obtained from the patients. This investigation was approved by the Ethics Committee of Tongji Hospital affiliated with Tongji University (Approval Number K2017005). This study was registered before patient enrollment at http://www.chictr.org.cn (ChiCTR-DDD-17010762). We obtained informed consent from every participant after a full protocol explanation. The authors followed the Declaration of Helsinki principles. This manuscript adheres to the applicable Strengthening the Reporting of Observational Studies in Epidemiology (STROBE) guidelines.

Eligibility criteria were as follows: (1) age > 65 years; (2) American Society of Anesthesiologists (ASA) Physical Status Classification I–III; and (3) participants were undergoing spinal stenosis decompression surgery.

The exclusion criteria: (1) pre-existing mental and/or psychiatric disease; (2) Parkinson’s disease (PD); (3) audition impairment, vision or language troubles impeding communication; (4) situations unsuitable for an MRI (claustrophobia); (5) diagnosis of dementia or previous stroke; (6) inability to undergo preoperative and postoperative cognitive evaluation; and (7) preoperative Mini-Mental State Examination (MMSE) < 20.

### Study Processes

**Figure 1:**
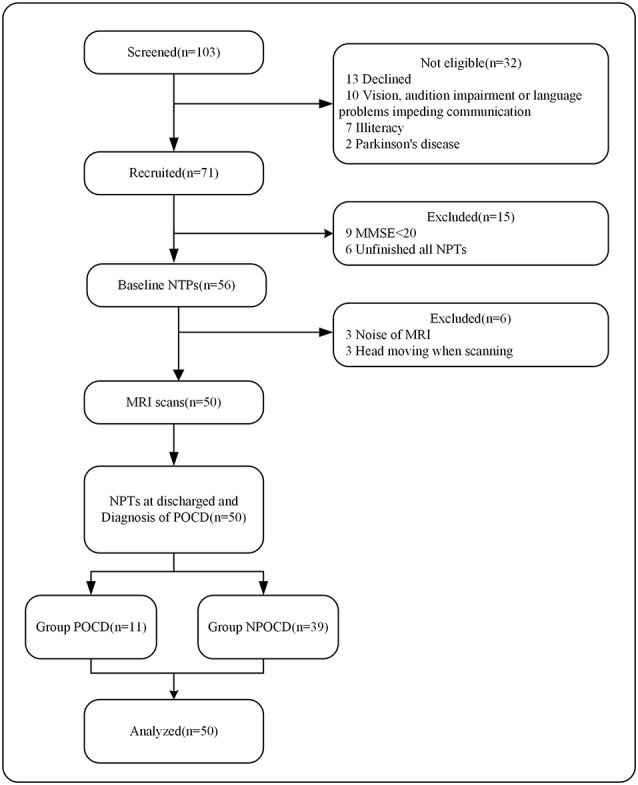
Flow diagram for study assessments. NPTs, neuropsychological tests; MRI, magnetic resonance imaging; POCD, postoperative cognitive dysfunction; NPOCD, non-POCD.

Study-related processes included intraoperative data collection, preoperative (baseline) multimodal assessments, and postoperative multimodal assessments of the study participants postoperatively at 1 week. Patients were recruited 1–3 days before surgery. The MRI scan and the baseline Neuropsychological Tests (NPTs) were performed one day before the surgery. Post-operative NPTs were performed on the 7th day of the surgery. To generate the control group mean and standard deviation (SD), the volunteers were examined in the same intervals as the patient group and the mean difference was calculated. The demographics, MRI data, NPTs results, pre- and post-operative data were collected and analyzed. Figure 1 illustrates the workflow of the investigation assessments.

### Neuropsychological Evaluation and POCD Definition

Neuropsychological evaluation was conducted by a well-trained neuropsychologist at two time points for patients and volunteers: (1) baseline: the day before surgery; and (2) 7 days postoperatively. In addition, subjects were first screened with MMSE and Montreal Cognitive Assessment (MoCA) to exclude subjects with cognitive impairment. Patients with an MMSE score <20 or who had Mild Cognitive Impairment (MCI) were not tested any further. Postoperative delirium was assessed daily by study members using either a disorder assessment (CAM) or a CAM-intensive care unit (CAM-ICU). Delirium assessments were completed daily until the 7th inclusive day of hospitalization.

The test battery consisted of 11 tests: the Rey Auditory Verbal Learning Test (AVLT immediate and delayed), Stroop Colour Word Test (STROOP), Digits Symbol Substitution Test (DSST), Judgment Of Line Orientation Test (JLOT), Brief Visuospatial Memory Test-revised (BVMT immediate and delayed), Trail Making Test (TMT), Semantic Fluency Test (SFT), and Forward And Backward Digit Span Test (DST forward and backward), which primarily focus on memory, attention, and executive function.

For assessing POCD, the difference between postoperative and preoperative test scores was used, instead of a single result. To generate a control group mean and SD, 20 healthy volunteers that were age and education-matched with the patients were tested in the same intervals as the patient group, and the mean difference was calculated. Improvement in the same repeated test scores is possible by subtracting the population mean from the observed differences in patient’s test scores to control the study effect. Healthy volunteers completed NPTs mentioned above at the same interval.

The difference in post- and pre-operative scores was termed x, and the counterpart in the volunteer group was μ (mean value for the difference in the volunteer group). The SD of the difference scores in volunteers was computed as δ, and the *Z*-score could be calculated as follows:

Z=(x−μ)/δ

POCD was defined as at least two NPT *|Z|*-scores ≥ 1.96.

### MRI Scan

All the subjects underwent MRI at Tongji Hospital using a 3.0-T MRI (Verio, Siemens, Erlangen, Germany) scanner equipped with a 32-channel head coil. In the MRI data acquisition, we instructed participants to stay awake, relax with their eyes closed, and remain motionless. Participants fasted for at least 4 h prior to the scan and withheld any medications with vasoactive properties on the day of the scan. Pulsed ASL(PASL) images were acquired using an echo-planar imaging (EPI) sequence with the following parameters: repetition time (TR) 3,500 ms, echo time(TE) 15 ms, flip angle 90°, slice thickness 3.5 mm without gap, field of view (FOV) 224 mm × 224 mm, matrix size 128 × 128, inversion time (TI_1_ 700 ms, TI_2_ 1800 ms), number of excitations 1, number of slices 33, total scan time 5 min and 31 s. ASL imaging protocol acquired an M_0_ image and 45 control/label pairs. High-resolution T1-weighted images were acquired using a whole brain three-dimensional brain volume imaging sequence(MPRAGE)with the following parameters: TR 2530 ms, TE 2.98ms, inversion time 450 ms, flip angle 7°, slice thickness 1 mm, no gaps, FOV 256 mm ×256 mm, matrix size 256 × 256, voxel size 1 mm ×1 mm ×1 mm, number of slices 192, total scan time 6 min and 3 s. For each subject, all images were inspected during the collection of MRI data to ensure that no visible artifacts were found. The artifact appeared in three of our subjects’ MR images, so the relevant sequence was rescanned immediately to obtain a qualified MR image of each of these subjects.

### Data Preprocessing

Imaging data were processed using the ASL toolbox (ASL tbx^1^; Wang et al., 2008) and SPM12.The brain imaging toolkit DPABI^2^ was used for data pre-processing and analysis(Yan et al., 2016). The detailed procedures have been described in a previous study (Wang et al., 2008). Steps to process ASL images included motion correction, spatial smoothing, exclude out-of-brain voxels, CBF quantization, partial volume correction, and spatial registration to Montreal Neurological Institute (MNI) standard brain space. Spatial smoothing was performed with an isotropic Gaussian kernel with FWHM = 6 mm. Pre-processed ASL labels and control image pairs were then successively subtracted. CBF was then calculated by the equation based on the M_0_ as follows:

(1) $$f=\frac{\lambda \Delta M}{2\alpha M_{0}TI_{1}\exp(-TI_{2}/T_{1\alpha })}$$

where *f* is regional CBF (in milliliters per 100 g per minute), λ is blood and tissue water partition coefficient which equals to 0.9 (in milliliters per gram), α is 95% inversion efficiency, M_0_ and ΔM are fully relaxed image intensity, and signal difference, respectively (control and label), TI_1_ and TI_2_ (in ms) are inversion times, and T_1α_ is 1,500 ms at 3 T (longitudinal relaxation time of blood; Xekardaki et al., 2015). The mean ASL image was registered to the high-resolution structural T1 image using SPM 12. The corresponding registration transform was used to register the CBF maps to the structural MRI.

With the help of segmentation tools of SPM12, the high resolution T1-weighted MRI was segmented into gray matter (GM), white matter (WM), and cerebrospinal fluid (CSF). The images obtained were then projected into local ASL image space, based on the registration correspondence between the mean ASL control image and the structural image, which was later used to extract the CBF signal for partial volume correction. The Diffeomorphic Anatomical Registration Through Exponential Lie Algebra (DARTEL) procedure tool in SPM12 was used to prompt a probable map of GM and WM for all the subjects based on their segmented local templates. By applying a linear affine transformation, the local template was warped to the MNI standard space. The individual subject’s brain was mapped into MNI space with these transformations. On each voxel of GM, a partial volume correction was carried out. As mentioned above, with the help of the same registration transformation from the mean ASL control image to the structural image, the partial volume corrected CBF maps were subsequently warped to the MNI space.

SPM12 software package was used to conduct statistical modeling and model evaluation of standardized cerebral perfusion images obtained by the above steps in the POCD group and NPOCD group. CBF maps in the two groups were compared using two-sample t-tests. To exclude individual differences, age, education, and gender were included as covariates in the regression. To control the false positive rate and reduce type I error, Gaussian random field (Gauss random field, GRF) principle of FWE was used for multiple corrections (FWE cluster—level corrected, *p* < 0.05, voxel—*p* < 0.001). Then, we got the statistically significant brain regions level.

Cluster obtained from the difference analysis between ASL groups was selected as region of interest (ROI), and the CBF value of ROIs was extracted as the average CBF of each region.

### Anesthetic Management

After obtaining written consent, standard anesthesia was provided. We induced anesthesia by sufentanil, etomidate, and cis-atracurium, followed by endotracheal intubation. We mechanically ventilated patients and held the end-tidal CO_2_ constant at 35 ± 5 mmHg. Following general anesthesia induction, we maintained patients with 1–1.2 minimal alveolar concentration sevoflurane with remifentanil and propofol TCI until the end of the surgery. All the patients were infused with cis-atracurium intermittently as required. During the entire surgical procedure, we rigorously monitored the electrocardiogram, arterial blood pressure, and oxygen saturation. We kept the bispectral index between 60 and 40 to guarantee appropriate anesthesia depth. We provided postoperative analgesia through patient-controlled analgesia.We delivered sufentanil at a rate of 1.5 μg/h, with a 1.5–2 μg bolus and lockout interval of 15 min for breakthrough pain. We assessed pain after surgery using the visual analog scale (VAS) score (0 = no pain and 10 = worst pain imaginable).

### Statistical Analysis

We performed statistical analysis using SPSS 21.0 (IBM, USA). Descriptive statistics of variables were examined in patients with and without POCD. Normally distributed data are presented as mean and standard deviation (SD), whereas non-normally distributed data are presented as median and inter-quartile range (IQR). Categorical data were expressed as the number and percentage. Standard Chi-square statistics or Fisher’s exact test was used to analyze categorical variables. Quantitative data were analyzed either using a two-sample t-test or Wilcoxon rank sum test when data deviated from the normal distribution.

## Results

### Enrolled Patients and Clinical Characteristics

A total of 71 patients were included. The trial details are shown in Figure 1. Fifty patients completed both the preoperative MRI scan and NPTs follow-up. The study group included 27 females and 23 males. We identified POCD in 11 of the 50 patients (22%) on the 7th day after surgery. No positive CAM scores occurred on the postoperative assessment. Demographic and operative data for these patients are presented in Tables s 1, 2, respectively based on the POCD presence or absence. The mean POCD patient age was 68 ± 2.4 years, while the mean NPOCD patient age was 71 ± 5.5 years. There was no significant difference in other clinical characteristics.

**Table 1:** Demographics.

	ALL (*n* = 50)	**POCD** (*n* = 11)	**NPOCD** (*n* = 39)	*P*-value
Demographics				
Age (years), mean (SD)	71 (5.1)	68 (2.4)	71 (5.5)	0.009*
Male sex (%)	23 (46)	5 (45)	18 (46)	0.529
Height (cm), mean (SD)	164.54 (8.5)	168.82 (9.6)	163.76 (7.7)	0.513
Weight (kg)	68.00	67.73	67.08	0.782
Median (IQR)	(62.38–72.50)	(59.00–77.00)	(63.00–73.00)	
Education (years)	8.94	8.36	9.10	0.605
Median (IQR)	(6.00–12.00)	(7.00–9.00)	(6.00–12.00)	
Comorbidities				
COPD (%)	1 (2.0)	0 (0.0)	1 (2.9)	0.284
Cardiopathy (%)	1 (2.0)	0 (0.0)	1 (2.9)	0.117
Diabetes (%)	7 (14)	2 (18)	5 (22.7)	0.396
Hypertension (%)	17 (50.0)	2 (28.5)	15 (55.5)	0.124

IQR, Interquartile Range; SD, Standard Deviation; COPD, Chronic Obstructive Pulmonary Disease. ^*^Represents t-test, p < 0.05..

**Table 2:** Clinical characteristics.

Intraoperative conditions	POCD (*n* = 11)	NPOCD (*n* = 39)	*p*-value
Surgical duration (min)
Mean (SD)	161.36 (49.55)	185.95 (57.58)	0.303
Anesthesia duration (min)
Mean (SD)	197.27 (44.46)	231.92 (63.70)	0.183
Bleeding volume (ml)	200.00	200.00	0.816
Median (IQR)	(100.00–300.00)	(100.00–325.00)
Systolic pressure (ml)	112.86	112.82	0.140
Mean (SD)	(5.37)	(9.36)
Diastolic pressure (ml)	73.00	69.43	0.862
Mean (SD)	(7.14)	(6.95)
Bispectral index	54.00	54.00	0.533
Median (IQR)	(51.00–56.00)	(53.00–56.00)
Visual analog score	2.29	2.21
Median (IQR)	(1.00–3.00)	(2.00–3.00)	0.118
Ramsay score	1.36	1.41
Median (IQR)	(1.00–2.00)	(1.00–2.00)	0.545

*IQR, interquartile range; SD, standard deviation*.

Table 3 shows the pre- and post-operative NPTs scores between the POCD group and the NPOCD group.

**Table 3:** Summary statistics of patients with and without POCD in different cognitive function tests.

NPTs	POCD (*n* = 11)	NPOCD (*n* = 39)
MMSE		
Preoperative, mean (SD)	28.57 (1.40)	27.46 (3.20)
Mo-CA		
Preoperative, mean (SD)	24.71 (3.45)	23.29 (4.55)
AVLT-I		
Preoperative, mean (SD)	19.00 (5.35)	17.29 (5.25)
Postoperative, mean (SD)	15.86 (5.43)	17.29 (6.46)
Difference, mean (SD)	−0.66 (4.91)	−2.83 (3.25)
AVLT-D		
Preoperative, mean (SD)	6.00 (3.36)	4.50 (3.18)
Postoperative, mean (SD)	3.83 (2.79)	4.61 (3.43)
Difference, mean (SD)*	1.21 (3.41)	−7.83 (1.76)
BVMT-I		
Preoperative, mean (SD)	16.00 (6.22)	14.29 (8.91)
Postoperative, mean (SD)	18.57 (8.36)	16.89 (9.23)
Difference, mean (SD)	−3.23 (4.43)	−3.26 (2.94)
BVMT-D		
Preoperative, mean (SD)	7.57 (3.05)	5.36 (4.43)
Postoperative, mean (SD)	7.50 (3.51)	5.93 (4.16)
Difference, mean (SD)	0.91 (7.28)	−1.52 (3.60)
DSST		
Preoperative, mean (SD)	35.43 (12.00)	26.86 (11.00)
Postoperative, mean (SD)	37.29 (13.07)	27.11 (10.80)
Difference, mean (SD)	0.78 (5.81)	0.83 (4.75)
TMT		
Preoperative, mean (SD)	63.71 (28.81)	83.68 (44.86)
Postoperative, mean (SD)	58.00 (27.80)	78.88 (42.85)
Difference, mean (SD)	0.36 (0.24)	0.38 (0.60)
DST-F		
Preoperative, mean (SD)	10.14 (3.13)	6.96 (2.50)
Postoperative,median (IQR)	9.00 (6.00–10.00)	7.00 (6.00–11.00)
Difference, mean (SD)	−0.20 (0.29)	−0.32 (0.24)
DST-B		
Preoperative, mean (SD)	6.57 (3.21)	4.54 (1.80)
Postoperative, mean (SD)	5.43 (2.44)	4.93 (1.65)
Difference, mean (SD)	0.13 (0.46)	−0.18 (0.37)
SFT		
Preoperative, mean (SD)	19.14 (6.20)	16.50 (4.97)
Postoperative, mean (SD)	20.00 (17.79)	17.71 (5.11)
Difference, mean (SD)	−0.20 (0.31)	−0.23 (0.80)
STROOP		
Preoperative, mean (SD)	46.43 (17.91)	34.61 (13.78)
Postoperative, mean (SD)	43.57 (20.96)	36.75 (17.139)
Difference, mean (SD)	−1.78 (0.95)	−2.22 (0.80)
JLOT		
Preoperative, mean (SD)	17.71 (1.70)	14.75 (2.84)
Postoperative, mean (SD)	15.14 (2.94)	15.37 (2.45)
Difference, mean (SD)*	−0.10 (1.98)	−2.07 (1.53)

*Difference is the cognitive change between preoperative and postoperative scores, which is displayed as Z-score of a single NPT. MoCA, Montreal Cognitive Assessment; MMSE, Mini-Mental State Examination; JLOT, Judgment Of Line Orientation Test; NPTs, Neuropsychological Tests; MRI, Magnetic Resonance Imaging; AD, Alzheimer’s disease; ASA, American Society of Anesthesiologists; SD, Standard Deviation; AVLT, Auditory Verbal Learning Test; AVLT-I, Auditory Verbal Learning Test-Immediate; AVLT-D, Auditory Verbal Learning Test-Delayed; Stroop, Stroop Colour Word Test; DSST, Digits Symbol Substitution Tests; BVMT, Brief Visuospatial Memory Test-Revised; BVMT-I, Brief Visuospatial Memory Test -Immediate; BVMT-D, Brief Visuospatial Memory Test-Delayed; TMT, Trail Making Test; SFT, Semantic Fluency Test; DST, Digit Span Test; DST-F, Digit Span Test-Forward; DST-B, Digit Span Test-Backward; SD, Standard Deviation; IQR, Interquartile Range*.

### MRI Results

**Figure 2:**
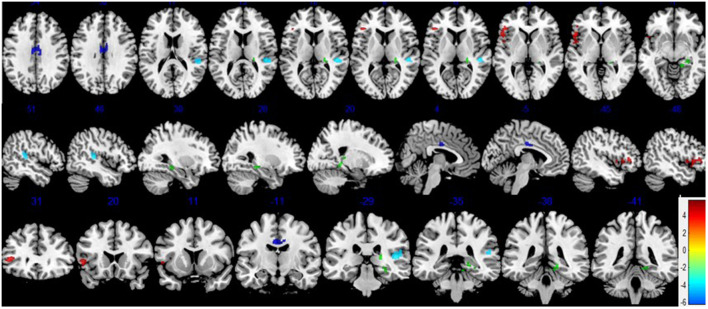
Differences in cerebral blood flow (CBF) between the POCD and NPOCD groups. Voxelwise comparison of the partial volume corrected ASL revealed decreased CBF in the right superior temporal gyrus of the temporal lobe, right and left middle cingulate gyrus, and the right hippocampus and the parahippocampal gyrus, and increased CBF in the pars triangularis of inferior frontal gyrus in POCD patients compared with NPOCD patients. Voxel-based comparison superimposed onto Montreal Neurologic Institute standard brain in the coronal, axial, and sagittal planes. Due to the large cluster of brain regions corrected by multiple comparisons, space is limited to show all sections of brain regions, and only some brain regions with statistical differences are shown. There was no significant difference in the overlapping brain regions. Blue to red color indicates low to high blood flow.

After FWE multiple comparison correction, we found significant CBF differences in brain areas including the frontal gyrus (the pars triangularis of inferior frontal gyrus), the temporal lobe (right superior temporal gyrus), the right hippocampus and the parahippocampal gyrus, and the cingulate gyrus (right and left medial gyrus). Hypoperfusion was observed in the right superior temporal gyrus of the temporal lobe, right and left middle cingulate gyrus, the right hippocampus, and parahippocampal gyrus in the POCD group. In addition, hyperperfusion in the pars triangularis of the inferior frontal gyrus in POCD patients were also found (Figure 2 and Table 4).

**Table 4:** Areas of hypoperfusion and hyperperfusion in the POCD compared to the NPOCD group.

Brain area	Cluster size	Peak MNI coordinate			Statistical value (T)
		*x*	*y*	*z*	
**Hyperperfusion**					
Frontal_Inf_Tri_L (aal)	246	−46	32	4	5.7251
**Hypoperfusion**					
Temporal Lobe					
Temporal_Sup_R (aal)	187	46	−30	8	−5.9962
Cingulate Gyrus					
Cingulum_Mid_R (aal)	209	−4	−1	34	−5.5871
Cingulum_Mid_L (aal)					
ParaHippocampal_R (aal)	149	24	−30	10	−5.7396
Hippocampus_R (aal)					

## Discussion

Cerebrovascular imaging has a great appeal in understanding neurological diseases. Among the various MRI parameters, ASL provides an indicator of tissue perfusion (Alsop et al., 2000, 2015; Johnson et al., 2005; Dai et al., 2009). Because MRI is a part of routine screening for cognitive decline in many centers, ASL is a cost-effective and operator-independent tool for assessing early cognitive decline that simply extends the existed scanning session by a few minutes. However, there have been limited studies using ASL MRI to investigate POCD. The primary goal of the current study was to explore the association between ASL-derived measures of brain CBF and POCD incidence. We found a significant association between CBF measures and POCD incidence in our study.

A large number of studies have shown that decreased CBF is a sensitive marker of early cognitive impairment (Scheff et al., 2015). We observed that the POCD subjects with normal performance evaluated by neuropsychological tests preoperatively displayed obvious decreased CBF both in a part of the temporal lobe, the cingulate cortex, the hippocampus, and parahippocampal gyrus, which might be interpreted as a neurocognitive reserve (CR; Tomlinson et al., 1968). Previous studies have found cognitive reserve in patients with AD, MCI, and Parkinson’s disease (PD; Bosch et al., 2010; Xekardaki et al., 2015). Cognitive reserve (CR) means individuals with a higher reserve are able to cope with brain pathology (i.e., decreased CBF in our study) through some form of active compensatory strategy better than those with lower reserve. CR can be defined as the ability to use alternate cognitive strategies, in order to optimize or maximize performance on cognitive tasks (Baldivia et al., 2008). The above so-called active compensatory strategy or alternative cognitive strategies include the individual predisposition, education, and social integration (Xekardaki et al., 2015). That is why some individuals may maintain normal cognitive function longer than other individuals. Therefore, CR provides a possible explanation for the current discrepancy between the evidence of brain damage and the absence of clinical symptoms (Stern, 2002). CR is hypothesized to moderate the association between brain pathology and the expression of the pathology rather than protecting the brain against the development of brain pathology (Brickman et al., 2011; Singh-Manoux et al., 2011). In other words, greater CR allows individuals to cope better with the cognitive changes associated with aging with reductive CBF in our study.Some individuals may maintain normal cognitive function longer than others due to their predisposition, education, and social integration. Subjects with POCD may initially be able to compensate for these changes of CBF and maintain normal cognitive status on account of the above reasons. Therefore, CBF may be a potential bio-marker of associated POCD. These may also indicate that surgery and anesthesia may accelerate the depletion of cognitive reserve in POCD patients, thus, cognitive symptoms may become apparent.

In our study, we found that POCD patients showed distinct decreased CBF preoperatively in the temporal lobe, the cingulate cortex, the hippocampus, and parahippocampal gyrus. The temporal lobe mainly functions as sensory information processor, turning it into meaningful memories, language, and emotions (Aminoff et al., 2013; Pauli et al., 2017). Previous studies have demonstrated hypoperfusion in the temporal lobe in early cognitive impairment patients (Pantoni, 2010; Goto et al., 2011; Maekawa et al., 2014). The cingulate cortex is involved in many brain diseases because of its diverse structure and behavioral functions, as well as its extensive connections to many different cortical regions involved in a variety of behaviors. This region is the center of emotion, behavior, and memory (Scheff et al., 2015). Yoshida et al. (2011) reported that in the early stage of AD, cingulate gyrus CBF decreased significantly. Xekardaki et al. (2015) demonstrated reduced ASL in the posterior cingulate cortex at baseline is associated with the development of subsequent subtle neuropsychological deficits in healthy elderly control subjects. Accumulating studies indicate that deterioration in cognitive function is associated with a reduction in local CBF in the hippocampus. Recently, a research demonstrated that CBF in the hippocampus decreases with the progression of AD (Li et al., 2020). Meanwhile, Another research found the strongest decrease in CBF in the left hippocampal region in the MCI in their recent study (Duan et al., 2020). These studies indicated that decreased cognitive function is associated with decreased regional CBF in the temporal lobe, the cingulate gyrus, the hippocampus, and parahippocampal gyrus, which may underline the role of decreased CBF in cognitive impairment, consistent with our results.

In contrast with the above, our results also showed that, compared with the NPOCD group, preoperative MRI in the POCD group indicated increased CBF in the frontal gyrus. Previous studies have demonstrated hypoperfusion in the frontal gyrus in patients with impaired cognitive function (Bangen et al., 2014; Ding et al., 2014; Zou et al., 2014; Zhang et al., 2021). There have also been some studies about hyperperfusion in the frontal gyrus in patients with impaired cognitive function which is consistent with our results (Duan et al., 2020). It was also found that reduced CBF can coexist with increased CBF in the early stages of a neurodegenerative process. It was suggested that hyperperfusion is a compensatory increase in neural activity (Alsop et al., 2008). Thus, we speculate that hyperperfusion in the frontal gyrus may occur in the POCD as a result of mechanisms in the brain that compensate for altered metabolism. Hypoperfusion occurs when these compensatory mechanisms fail at later stages of the disease process. This may provide further evidence for the existence of compensatory mechanisms in POCD patients at an early age.

Based upon the former and current results, we speculated that these patients have no clinically detectable cognitive symptoms due to the cognitive reserve and/or compensatory mechanism, whereas cognitive symptoms would become apparently deteriorated after stressors such as surgery and anesthesia. Therefore, we can identify high-risk patients with POCD earlier through preoperative MRI results for early prediction and prevention.

More interestingly, we found that patients in the POCD group were even less elderly than those in the NPOCD group, inconsistent with previous studies (Czyz-Szypenbejl et al., 2019), further indicating that MRI detection was more sensitive and accurate than the age prediction of POCD.

The main limitation of the current study was the small sample size. The difficulty of patient recruitment may have led to a relatively moderate sample size, which could lead to potential type II errors. The second limitation was that we had only 7 days of follow-up. We could not conduct longer postoperative follow-up to help us determine the long-term prognosis of patients. Therefore, the correlation between preoperative MRI examination and long-term cognitive impairment needs to be further elucidated. Finally, the current study measured regional CBF using PASL which has lower signal- to-noise ratio than the recommended pseudo-continuous ASL with background suppression. With the improvement of equipment and research technology, we will provide better experimental results in further studies.

## Conclusion

The current study showed the difference in preoperative CBF in the frontal gyrus (the pars triangularis of inferior frontal gyrus), the temporal lobe (right superior temporal gyrus), the right hippocampus and parahippocampal gyrus, and the cingulate gyrus (right and left medial gyrus) in POCD compared with NPOCD patients. These results suggested that pre-operative CBF might be a more sensitive bio-marker of patients who developed early POCD after major non-cardiac surgery. Based upon former and present results, we speculated that POCD patients would have subtle cognitive decline and MRI alterations before surgery. Thus, we can diagnose patients at high risk of POCD with preoperative MRI results for early prediction and prevention.

## Data Availability Statement

The raw data supporting the conclusions of this article will be made available by the authors, without undue reservation.

## Ethics Statement

The studies involving human participants were reviewed and approved by Ethics Committee of Tongji Hospital affiliated with Tongji University (Approval Number K2017005). The patients/participants provided their written informed consent to participate in this study. Written informed consent was obtained from the individual(s) for the publication of any potentially identifiable images or data included in this article.

## Author Contributions

XD drafted the manuscript. YG performed the MRI scan and ASL data analysis. Jianhui Liu designed the protocol. PW, XD, SL, JY, YD, JH, JZ, and Jiehui Liu acquired the data. XD, YG, and Jianhui Liu analyzed the data. DB refined the language. All authors contributed to the interpretation of the data and revision of the manuscript. All authors contributed to the article and approved the submitted version.

## Conflict of Interest

The authors declare that the research was conducted in the absence of any commercial or financial relationships that could be construed as a potential conflict of interest.

## Publisher’s Note

All claims expressed in this article are solely those of the authors and do not necessarily represent those of their affiliated organizations, or those of the publisher, the editors and the reviewers. Any product that may be evaluated in this article, or claim that may be made by its manufacturer, is not guaranteed or endorsed by the publisher.

## Footnotes

^1^ http://cfn.upenn.edu/~zewang

^2^ http://www.restfmri.net
